# Authors’ Response to Peer Reviews of “Impact of Modifiable Risk Factors on the Occurrence of Cutaneous Leishmaniasis in Diyala, Iraq: Case-Control Study”

**DOI:** 10.2196/31512

**Published:** 2021-07-30

**Authors:** Asaad Mahdi Lehlewa, Hanan Abdulghafoor Khaleel, Faris Lami, Saif Aldeen Falah Hasan, Hasanain Asmail Malick, Razzaq Hashim Mohammed, Qais Abdulazziz Abdulmottaleb

**Affiliations:** 1 Public Health Directorate Ministry of Health of Iraq Baghdad Iraq; 2 Communicable Diseases Control Center Public Health Directorate Ministry of Health of Iraq Baghdad Iraq; 3 Department of Community and Family Medicine College of Medicine University of Baghdad Baghdad Iraq; 4 Ministry of Health of Iraq Baghdad Iraq; 5 Ministry of Health of Iraq Babylon Iraq

**Keywords:** cutaneous leishmaniasis, outbreak, Iraq, risk factors, risk, disease, infectious disease, disease prevention, prevention

*This is the authors’ response to peer reviews of “Impact of Modifiable Risk Factors on the Occurrence of Cutaneous Leishmaniasis in Diyala, Iraq: Case-Control Study.”*

## Round 1 Review

### Reviewer O

1. Thanks for raising this issue [1]. Being a control does not mean that they should not share the risk factors. A control here [2] is a person with no current lesions or history of lesions.

4. Would you please highlight the repeated paragraph, as we reviewed it more than once but did not find it.

### Reviewer T

2. Because we have so many risk factors to talk about, we did not put the odds in the Abstract.

6. Because this study was part of an outbreak investigation, we did not use the traditional methods for sample size calculation. Instead, we included as many as possible of both cases and controls to have more insight into the risk factors.

8. If the reviewer can kindly point out which table he meant [3], we will correct it accordingly.

## References

[1] Al-Dubaiee R (2021). Peer review of "Impact of modifiable risk factors on the occurrence of cutaneous leishmaniasis in Diyala, Iraq: case-control study". JMIRx Med.

[2] Lehlewa AM, Khaleel HA, Lami F, Hasan SAF, Malick HA, Mohammed RH, Abdulmottaleb QA (2021). Impact of modifiable risk factors on the occurrence of cutaneous leishmaniasis in Diyala, Iraq: case-control study. JMIRx Med.

[3] Nassar, AAH (2021). Peer review of "Impact of modifiable risk factors on the occurrence of cutaneous leishmaniasis in Diyala, Iraq: case-control study". JMIRx Med.

